# Danzhi Jiangtang Capsule Mediates NIT-1 Insulinoma Cell Proliferation and Apoptosis by GLP-1/Akt Signaling Pathway

**DOI:** 10.1155/2019/5356825

**Published:** 2019-07-29

**Authors:** Yuan-Jie Wu, Yuan-Bo Wu, Zhao-Hui Fang, Mei-Qiao Chen, Yu-Feng Wang, Chuan-Yun Wu, Ming-An Lv

**Affiliations:** ^1^Department of Basic Theory of Chinese Medicine, Anhui University of Chinese Medicine, Hefei 230012, China; ^2^Department of Neurology, Anhui Provincial Hospital, Anhui Medical University, Hefei 230001, China; ^3^Department of Neurology, Anhui Provincial Hospital, The First Affiliated Hospital of University of Science and Technology of China, Hefei 230001, China; ^4^Department of Endocrinology, the First Affiliated Hospital of Anhui University of Chinese Medicine, Hefei 230031, China

## Abstract

**Objective:**

This study aimed to investigate the effects of Danzhi Jiangtang Capsule (DJC) on the proliferation and apoptosis functions of NIT-1 pancreatic *β*-cells exposed to high-glucose load through GLP-1 activated Akt/ FoxO1 signaling pathway.

**Methods:**

Cellular apoptosis of NIT-1 pancreatic *β*-cells was induced by culturing in medium with 33.3mmol/L high glucose (HG). Then low-dose DJC (HG +LD), high-dose DJC (HG +HD), high-dose DJC+ GLP-1 inhibition (HG +HD +GI), and high-dose DJC+AKT inhibition (HG +HD+AI) were added, respectively. Cellular proliferation was accessed by cell counting kit (CCK-8) and cellular apoptosis was measured by Annexin V-FITC/PI staining. The protein levels of phosphorylated phosphatidylinositol-3-kinase (p-PI3K), phosphorylated AKT (p-AKT), phosphorylated Forkhead box protein O1 (p-FoxO1), and cleaved caspase-3 were detected by Western blotting. The mRNA expression of pancreatic duodenal homeobox-1 (PDX-1), CyclinD1, Bcl-2, and insulin was tested by Q-PCR.

**Results:**

Comparing to HG group, (HG+HD) group showed a significantly increased cellular proliferation. The apoptosis of NIT-1 cells also was obviously reduced, with downregulated cleaved caspase-3 protein level and upregulated PDX-1, CyclinD1, and Bcl-2 mRNA levels (P<0.05). Additionally, (HG+HD) group manifested increased insulin mRNA expression; the protein levels of p-PI3K and p-AKT were markedly increased and p-FoxO1 was decreased. All of the above therapeutic effects by DJC intervention had been reversed by GLP-1 inhibition in (HG+HD+GI) group or AKT inhibition in (HG+HD+AI) group.

**Conclusion:**

DJC was able to attenuate the toxicity of high-glucose load in NIT-1 pancreatic *β*-cells, ascribed to the improvement of cellular proliferation and apoptosis by GLP-1/Akt signaling pathway. This study could supply a new mechanism of DJC effects on type 2 diabetes mellitus (T2DM) treatment.

## 1. Introduction

The crucial characteristics of type 2 diabetes mellitus (T2DM) are pancreatic *β*-cell dysfunction initially and insulin resistance finally [1]. Hyperglycemia has been proved to induce loss of pancreatic *β*-cell [2–4]. Long-term exposure to high-glucose load can induce glucotoxicity, which leads to deterioration of *β*-cell function in many experiments via endoplasmic reticulum stress, oxidation stress, and vasoactive cytokine activation [5–7]. Many evidences have shown that pancreatic *β*-cells dysfunction is a central part of the T2DM progression and *β*-cells play an important role to maintain glucose metabolism balance via activated insulin secretion [8, 9]. Hence, exploring the underlying mechanisms of *β*-cell dysfunction may attribute to new clinical treatment strategies.

The compound preparation of Danzhi Jiangtang Capsule (DJC) is for reinforcing Qi, nourishing Yin, and promoting blood circulation to remove blood stasis; it is composed of radix pseudostellariae, dried rehmannia root, semen cuscutae, moutan bark, rhizoma alismatis, and leech. DJC has been used for T2DM treatment for years and both clinical and laboratorial studies have reported its beneficial effects on T2DM. In elderly T2DM patients, DJC could modify *β*-cell function [10]. Moreover, its effect of decreasing blood glucose in T2DM patients has been found [11]. In T2DM rat models, DJC combined with exercise attenuated oxidative stress and *β*-cell injury [12]. However, the mechanisms of DJC protection effects on *β*-cell remain unclarified.

The physiological functions of glucoincretin hormone glucagon-like peptide-1 (GLP-1) are to stimulate insulin biosynthesis and secretion in T2DM patients [13]. In cultured pancreatic *β* (INS-1) cells, GLP-1 acted as a cellular growth factor through activating phosphatidylinositol 3-kinase (PI3K)/Akt signaling pathway [14]. Forkhead box protein O1 (FoxO1) is a signal molecule of insulin signaling pathway downstream, and its activity is suppressed by phosphorylation of PI3K /Akt pathway, leading to pancreatic *β*-cell mass increase [15, 16]. In this study, we explored the effect of DJC on NIT-1 pancreatic *β*-cell function and the potential mechanism of GLP-1/ Akt signaling pathway.

## 2. Methods and Materials

### 2.1. Mouse NIT-1 Pancreatic *β*-cell Culture

Mouse NIT-1 pancreatic *β*-cells were purchased from Tongji Medical College of Huazhong University of Science and Technology (Wuhan, China). Cells were cultured in RPMI 1640 medium (Gibco, USA) containing 10% fetal bovine serum (Sigma, USA), for 37°C and 5% CO_2_. For cells growing near fusion, there was 0.25% trypsin digestion (Gibco, USA) for subculture.

### 2.2. The Preparation of Medicated Serum

The DJC was made by the First Affiliated Hospital of Anhui Medical University (Batch number: Z20090006, patent number. ZL200310112845.1), and the dosage for adults was 6g/d. According to the conversion method of body surface area, the high dose of DJC for rats was 1.26g/(kg·d), the low dose of DJC for rats was 0.63g/(kg·d), and the DJC was solved in 5ml normal saline (NS), two times each day. 30 SD rats were randomly divided into control group, high dose of DJC group, and low dose of DJC group; each group contained 10 rats. Both groups were treated for 7 days. 2 h after last treatment, rats were anaesthetized by sodium pentobarbital (Sigma, USA), blood was collected from abdominal aorta, serum was separated under sterile condition, inactivated in 56°C water for 30min, then the medicated serums were saved in - 80°C for later use.

### 2.3. Experimental Groups and Interventions

Mouse NIT-1 pancreatic *β*-cells growing near fusion were subcultured in 96-wells plate and then stimulated with 33.3mmol/L high glucose (HG). Then they were added low-dose DJC (HG +LD), high-dose DJC (HG +HD), high-dose DJC+ GLP-1 inhibition (HG +HD +GI), and high-dose DJC+AKT inhibition (HG +HD+AI) respectively. Exendin9-39 was used as GLP-1 receptor inhibition, 1*∗*10^−5^ mmol·L^−1^ and AKT inhibition (MK-2206 2HCl, Selleckchem, Houston, TX, USA), 5umol·L^−1^. The medicated serums were added for coculturing 24h; then, Exendin9-39 or AKT inhibition was added for another 24h. Mouse NIT-1 pancreatic *β*-cells normally cultured were used as control (CTL) group.

### 2.4. Cell Counting Kit (CCK-8) Assay

Mouse NIT-1 pancreatic *β*-cells growing near fusion were subcultured in 96-well plates, 100ul per well; after culturing for 24h, each well was added 10ul CCK-8 solution (Sigma, USA), with 37°C and 5% CO_2_ for 1-4h. The optical density (OD) was measured under 450nm.

### 2.5. Annexin V-FITC/PI Apoptosis Detection

Mouse NIT-1 pancreatic *β*-cells growing near fusion were subcultured in 6-well plates, given different interventions. Then cells were washed with PBS and digested by Trypsin-EDTA (0.05%), washing twice using 4°C PBS, making liquid suspension cells 400ul, then adding 5 *μ*l Annexin V-FITC staining fluid (Sigma, USA), blending lucifugal in 4°C for 15 min, 10ul PI staining fluid (Sigma, USA), blending lucifugal in 4°C for 5 min. The cellular apoptosis was tested by flow cytometry. Upper left quadrant referred to dead cells, upper right quadrant referred to apoptosis cells at late stage, lower left quadrant referred to normal cells, and lower right quadrant referred to apoptosis cells at early stage.

### 2.6. Western Blotting Analysis

Protein concentrations were determined using a BCA assay kit (Thermo, USA). Antibodies against PI3K (Abcam, USA, catalog: ab151549), p-PI3K (Abcam, USA, catalog: ab182651), Akt (Abcam, USA, catalog: ab179463), p-Akt (Abcam, USA, catalog: abab131443), FoxO1 (Abcam, USA, catalog: ab39670), p-FoxO1 (Abcam, USA, catalog: ab131339) and caspase-3 (Abcam, USA, catalog: ab13847USA), Histone H3(Abcam, USA, catalog: ab239403), and beta-actin (Abcam, USA, catalog: ab8226) were used to identify specific proteins, which were visualized by ECL method. Image J software was used for grey value statistics.

### 2.7. Total RNA Preparation and Real-Time PCR

Extracting total RNA in the NIT-1 cells, 5*μ*l RNA was used for 1% agarose gel electrophoresis (AGE) to detect the integrity of the RNA. Measuring the optical density (OD) value at 260nm and 280nm was used to calculate the RNA content and purity. There was reverse transcription of RNA to synthetize cDNA and fluorescent quantitative PCR was executed for 45 rounds (95°C 10min, 95°C 15s, 60°C 60s). Primer sequences were PDX-1 forward: 5′-ACTTAACCTAGGCGTCGCAC-3′, reverse: 5′- AGCTCAGGGCTGTTTTTCCA -3′; CyclinD1 forward: 5′- TCAAGTGTGACCCGGACTG -3′, reverse: 5′- ATGTCCACATCTCGCACGTC -3′; Bcl-2 forward: 5′- GAACTGGGGGAGGATTGTGG -3′, reverse: 5′- GCATGCTGGGGCCATATAGT-3′; insulin forward: 5′-ACGAACACTTTGCCATTGCC-3′, reverse: 5′- CCTTTGCCCGATTATGCAGC-3′; actin forward: 5′- GCCCTGAGGCTCTCTTCCA-3′, reverse: 5′- GCGGATGTCGACGTCACA-3′.

### 2.8. Statistical Analysis

GraphPad Prism software 6.0 was used for data analysis. The data were presented as Mean ± SD. A two-sided student t-test was used to examine individual differences when comparing two groups, and a one-way ANOVA was used to examine individual differences when comparing three groups. P < 0.05 was considered to be statistically significant.

## 3. Results

### 3.1. Effect of DJC on Cellular Proliferation in NIT-1 Cells

**Figure 1:**
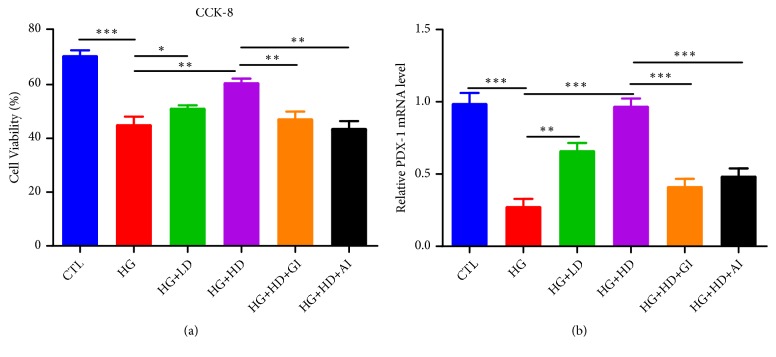
*The cellular proliferation in NIT-1 cells*. NIT-1 cells were cultured with rats' serums containing high dose or low dose of DJC for 24h; then, they were additionally treated with 1*∗*10^−5^ mmol·L^−1^ GLP-1 receptor inhibition or 5umol·L^−1^ AKT inhibition for another 24h. (a) The cell viability assay using CCK-8 kit; (b) Q-PCR analysis of PDX-1 mRNA level changes. Data were shown as Mean ± SD. Each group was independently repeated for three times. CTL, control; HG, high glucose; LD, low dose of DJC; HD, high dose of DJC; GI, GLP-1 receptor inhibition; AI, AKT inhibition. ^*∗*^*p <0.05, *^*∗∗*^*p<0.01, *^*∗∗∗*^*p<0.001*.

As shown in Figure 1, high-glucose load led to significant reduction of cellular proliferation in NIT-1 cells, measuring by CCK-8 assay (Figure 1(a)) and mRNA level of PDX-1 (Figure 1(b)). Additional low-dose of DJC intervention could attenuate the impaired cellular proliferation induced by high glucose, and this beneficial effect had been amplified when the dose of DJC increased. However, treatment effect of DJC had been blocked by either GLP-1 inhibition or Akt inhibition.

### 3.2. DJC Improved High-Glucose Induced Reduction of Insulin mRNA Level in NIT-1 Cells

**Figure 2:**
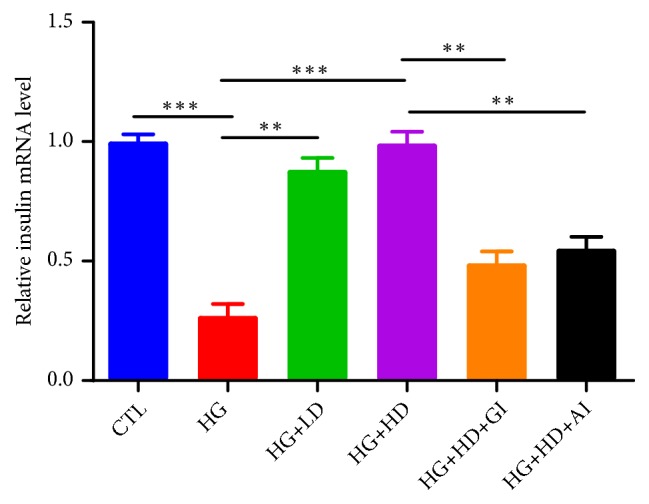
*The mRNA level of insulin in NIT-1 cells*. NIT-1 cells were cultured with rats' serums containing high dose or low dose of DJC for 24h; then, they were additionally treated with 1*∗*10^−5^ mmol·L^−1^ GLP-1 receptor inhibition or 5umol·L^−1^ AKT inhibition for another 24h. Each group was independently repeated for three times. Data were shown as Mean ± SD. ^*∗*^*p<0.05, *^*∗∗*^*p<0.01, *^*∗∗∗*^*p<0.001*.

Figure 2 presented the mRNA levels of insulin in each group. High-glucose load could reduce the mRNA expression level of insulin in NIT-1 cells, and DJC treatment markedly increased the mRNA level of insulin, which had been reversed by either GLP-1 inhibition or Akt inhibition.

### 3.3. Effect of DJC on Cellular Apoptosis in NIT-1 Cells

**Figure 3:**
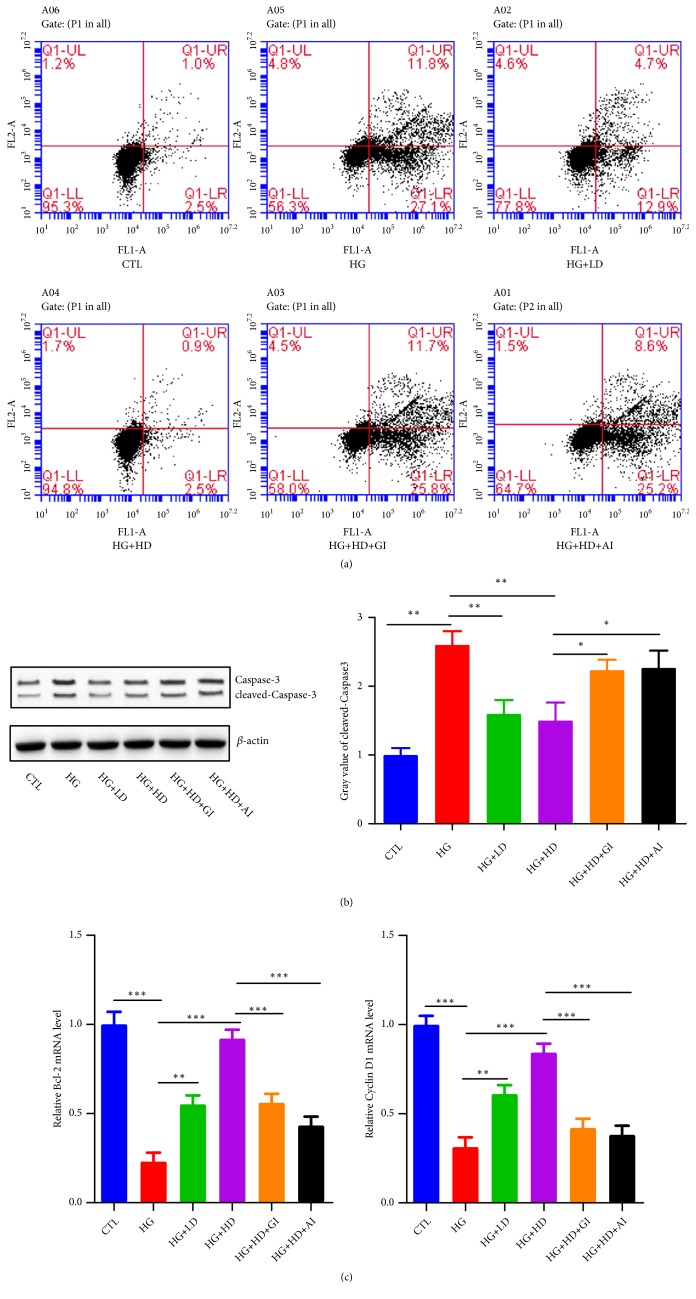
*The cellular apoptosis in NIT-1 cells*. NIT-1 cells were cultured with rats' serums containing high dose or low dose of DJC for 24h; then, they were additionally treated with 1*∗*10^−5^ mmol·L^−1^ GLP-1 receptor inhibition or 5umol·L^−1^ AKT inhibition for another 24h. (a) Annexin V-FITC/PI staining for flow cytometry sorting, the upper left quadrant (UL) represented dead cells, left lower quadrant (LL) represented normal cells, upper right quadrant (UR) represented early apoptosis cells, the lower right quadrant (LR) represented the late apoptotic cells; Apoptosis%=UR+LR; (b) the protein levels and grey value statistics of caspase and cleaved caspase-3; (c) Q-PCR analysis of Bcl-2 and Cyclin D1 mRNA level changes. Each group was independently repeated for three times. Data were shown as Mean ± SD. ^*∗*^*p<0.05, *^*∗∗*^*p<0.01, *^*∗∗∗*^*p<0.001*.

The total proportion of apoptosis NIT-1 cells at both early and late stage in HG group was (27.1%+11.8%)=38.9%; it was obviously higher than CTL group (Figure 3(a)). In (HG+LD) group, the apoptosis cell proportion was decreased to (12.9%+4.7%)=17.6%. Moreover, it continued to fall to (2.5%+0.9%)=3.4% in (HG+HD) group. The protein levels of cleaved caspase-3 showed similar changes according to different interventions (Figure 3(b)). Bcl-2 is one of key regulators of apoptosis; knockout of Bcl-2 gene in mice showed more cellular apoptosis [17]. In our study, the mRNA level of Bcl-2 in HG group was markedly higher than CTL group, and DJC treatment decreased the Bcl-2 mRNA expression. PDX-1, mainly expressed in *β*-Cell, plays an important role in cellular survival specific genes insulin related gene expression [18].The mRNA level of PDX-1 had similar change with Bcl-2 (Figure 3(c)). The effect of DJC on cellular apoptosis reduction in NIT-1 cells could be cancelled by either GLP-1 inhibition or Akt inhibition.

### 3.4. Effect of DJC on GLP-1/Akt Signaling Pathway in NIT-1 Cells

**Figure 4:**
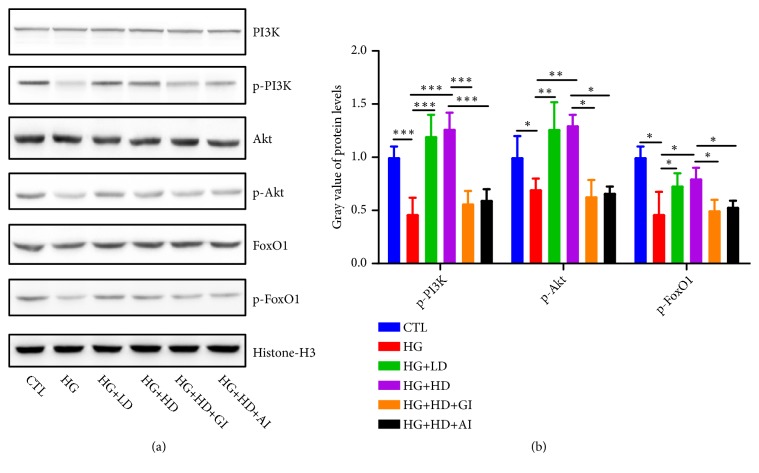
*The protein levels of GLP-1/Akt signaling pathway molecules*. NIT-1 cells were cultured with rats' serums containing high dose or low dose of DJC for 24h; then, they were additionally treated with 1*∗*10^−5^ mmol·L^−1^ GLP-1 receptor inhibition or 5umol·L^−1^ AKT inhibition for another 24h. (a) The protein levels of PI3K/p-PI3K, Akt/p-Akt, FoxO1/p-FoxO1 in each group; (b) the grey value statistics of p-PI3K, p-Akt, and p-FoxO1. Data were shown as Mean ± SD. ^*∗*^*p<0.05, *^*∗∗*^*p<0.01, *^*∗∗∗*^*p<0.001*. (1) S.K. JIANG Zaifang, Shen Ying. “Zhu Futang Practice of Pediatrics, 8th ed,”* People*'*s Medical Publishing House*.

As shown in Figure 4(a), the protein expression levels of p-PI3K, p-Akt, and p-FoxO1 were significantly downregulated in HG group, compared to CTL group. Treatment of DJC could upregulate the protein expression levels of p-PI3K, p-Akt, and p-FoxO1, which had been blocked by GLP-1 inhibition or AKT inhibition. These evidences gave great support to the hypothesis that the effects of DJC on NIT-1 cells were through GLP-1/Akt signaling pathway.*Figure 4(b) showed the grey value statistics of* p-PI3K, p-Akt, and p-FoxO1 in each group.

## 4. Discussion

The prevalence of T2DM has been rising each year, which is paralleled to the increase of population with high risk for diabetic development [19]. DJC had been reported to have potential hypoglycemic effects and the efficacy of improving insulin resistance was confirmed in both vivo and vitro experiments [12, 20]. Nevertheless, few studies reported the treatment of DJC on pancreatic *β*-cells with high-glucose exposure.

Increasing evidences showed that sustainably exposed to high-glucose load led to the decrease of insulin storage and insulin related gene levels in pancreatic INS-1 cells [9]. In our study, we used 33.3mmol/L concentration as a high-glucose stimulation; the expression of insulin mRNA in INS-1 cells was reduced after high-glucose load. Due to the unclarified pharmacokinetics of DJC, we used rats' serum which was treated with high dose and low dose of DJC to validate the mechanism of DJC in vitro studies. We found that the DJC intervention had an improved effect on insulin mRNA level; moreover, the DJC upregulated the insulin mRNA in a dose-dependent manner. According to these data, we concluded that DJC could improve the impaired insulin mRNA levels in the INS-1 pancreatic *β*-cells under high-glucose load. Using rats' medicated serum for the research made it difficult to exclude the influences of something else such as inflammatory factors, cytokines, and oxidative stress level in serum, so we could not say the effects of DJC were direct or indirect. Further investigation should be executed to stimulate INS-1 pancreatic *β*-cells with different concentrations of DCJ solution directly. The optimal dose for the best treatment effect needs further investigation.

Some studies indicated that insulin mRNA is activated by PDX-1 [21]. Sustainably exposure to high-glucose load could decrease the level of PDX-1 [22], which was consistent with our results. The data showed that the mRNA level of PDX-1 was markedly downregulated by high-glucose load. There was no relevant study to report the effect of DJC on PDX-1 expression level in pancreatic *β*-cells. Our data firstly showed that DJC intervention significantly upregulated the expression level of PDX-1. PDX-1 also played an important role in cellular survival specific genes in *β*-cells [18]. The PDX-1 mRNA reduction suggested the decline of cell vitality, which presented in our data by decreased cellular proliferation and increased cellular apoptosis, and DJC intervention could improve cell vitality.

We hypothesized that DJC may improve the protein expressions of insulin signal pathway impaired by high-glucose load in INS-1 cells. Insulin initially binds its cell-surface receptor subunit and then activates the intracellular tyrosine kinase domain. The insulin receptor substrate (IRS) has been phosphorylated and regulates downstream molecular of PI3K/AKT [23]. Our findings showed that DJC increased the phosphorylation of PI3K, AKT and downstream FoxO1 in INS-1 cells with dysfunction stimulated by high-glucose load. Furthermore, all these effects could be reversed by GLP-1 inhibition or Akt inhibition. We also tested the GLP-1 levels of rats serums in each group, and we could not find the significant difference between each group (P>0.05), so we judged that the effects of DJC were through activating GLP-1 pathway but not through increasing GLP-1 levels. From all above data, we concluded that DJC cloud attenuate the toxicity of high-glucose load in NIT-1 pancreatic *β*-cells, ascribed to the improvement of cellular proliferation and apoptosis by activation of GLP-1/Akt signaling pathway.

## 5. Conclusion

This study showed strong evidence that DJC could improve the impaired insulin mRNA expression, cellular proliferation and apoptosis induced by high-glucose load in INS-1 pancreatic *β*-cells. The underlying mechanism for these effects was potentially attributed to activating of the GLP-1/PI3K/Akt/FoxO1 pathway. Additionally, the effects of DJC on high-glucose induced INS-1 cells injury could be reversed by GLP-1 inhibition or Akt inhibition. Therefore, DJC can be an effective therapy in T2DM patients and deserves further investigation.

## Data Availability

The data used to support the findings of this study are available from the corresponding author upon request.
